# Composition and Antioxidant Activities of Volatile Organic Compounds in Radiation-Bred *Coreopsis* Cultivars

**DOI:** 10.3390/plants9060717

**Published:** 2020-06-04

**Authors:** Bo-Ram Kim, Hyun Mi Kim, Chang Hyun Jin, Si-Yong Kang, Jin-Baek Kim, Yeo Gyeong Jeon, Kong Young Park, Ik-Soo Lee, Ah-Reum Han

**Affiliations:** 1 Advanced Radiation Technology Institute, Korea Atomic Energy Research Institute, Jeongeup-si, Jeollabuk-do 56212, Korea; boram1606@kaeri.re.kr (B.-R.K.); khm2172@kaeri.re.kr (H.M.K.); chjin@kaeri.re.kr (C.H.J.); sykang@kaeri.re.kr (S.-Y.K.); jbkim74@kaeri.re.kr (J.-B.K.); 2 College of Pharmacy, Chonnam National University, Gwangju 61186, Korea; 3 Uriseed Group, Icheon-si, Gyeonggi-do 17408, Korea; ygjeon@uriseed.com (Y.G.J.); uriseeds@naver.com (K.Y.P.)

**Keywords:** *Coreopsis rosea*, Asteraceae, volatile compound, antioxidant activity

## Abstract

*Coreopsis* is a flowering plant belonging to the Asteraceae family. It is an ornamental plant native to the Americas, Asia and Oceania and its flower is used as a raw material for tea and food manufacture in China. In this study, new cultivars of *C. rosea* (“golden ring”) were developed via radiation-induced mutation of the original cultivar, “pumpkin pie”. The chemical composition and antioxidant activities of flowers belonging to three different *Coreopsis* cultivars were evaluated: “golden ring”, “pumpkin pie” and “snow chrysanthemum” (coreopsis tea; *C. tinctoria*). The volatile compounds were characterized via gas chromatography-mass spectrometry (GC-MS) and 50–59 oils representing 95.3–96.8% of the total volatile compounds in these flower materials were identified. ”Golden ring” contained a high amount of fatty acids (38.13%), while “pumpkin pie” and “snow chrysanthemum” teas were rich in aliphatic amides (43.01%) and esters (67.22%), respectively. The antioxidant activities of the volatile oils of these cultivars were evaluated using 1,1-diphenyl-2-picrylhydraxyl (DPPH) and 2,2-azino-bis-3-ethylbenzothiazoline-6-sulfonic acid (ABTS) radical scavenging assays. The volatile extract of “golden ring” showed higher antioxidant activities compared with the extracts of the other cultivars. Therefore, “golden ring” can be used for further development as a raw material for tea manufacture or as a dietary supplement.

## 1. Introduction

Approximately 80 species of *Coreopsis* (Asteraceae) are native to North America and are currently distributed throughout the Americas, Asia and Oceania [1]. *Coreopsis* thrives as annual and perennial types, and is an ornamental plant used to decorate gardens and roadsides [1,2]. In addition, the ethnopharmacological use of this plant’s flower has been reported [3]. It has been traditionally used to treat diarrhea, vomiting, and bleeding in North America [3]. The *Coreopsis* flower has been consumed as a drink to control diabetes in China and Portugal [3]. Currently, the flowers of *C. tinctoria*, known as “snow chrysanthemum”, are used as tea, with detoxifying and cooling effects, in China [4,5]. In a previous phytochemical study of *Coreopsis* species, phenolics, flavonoids (aurones, chalcones, and flavones), acetylenes and volatile oils were reported [2,3,4,5,6,7,8,9]. The plant’s extracts or constituents have been found to exhibit diverse biological activities such as antileukemic [2], antidiabetic [3,7], antioxidant [5,6], anti-inflammatory [7,8] and chemopreventive effects [9].

Hybridization between plants and mutations has yielded numerous varieties with high crop productivity and improved quality [10,11]. Mutation breeding via spontaneous mutation, ultraviolet light, chemical mutagenesis and ionizing radiation can be used effectively to induce genetic diversity [11]. Approximately 50% of the mutant varieties registered in the joint Food and Agriculture Organization/International Atomic Energy Agency (FAO/IAEA) mutant variety database are mutants induced by gamma irradiation [12]. *Coreopsis* species are of little commercial importance, but gardeners’ preferences for them are increasing due to their cold resistance, attractive flowers and leaves and tolerance of various environmental conditions [13]. Therefore, our research group has developed a new mutant cultivar of *C. rosea* (“golden ring”) bred via the exposure of stem cuttings of the original cultivar, “pumpkin pie”, to gamma irradiation and registered it in the Korea Seed and Variety Service [14]. *C. rosea* has been mainly studied for breeding and culture growth [15]; however, no studies have reported its chemical constituents and pharmacological activity until now.

**Figure 1:**
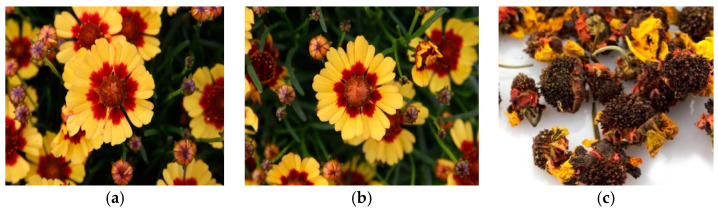
The flowers of (**a**) the gamma-irradiated mutant cultivar, “golden ring”; (**b**) its original cultivar, “pumpkin pie”; (**c**) the commercially available tea material, “snow chrysanthemum” (photographed by authors, Y.G.J. and B.-R.K.).

In this study, we analyzed the volatile oils of flowers obtained from a new cultivar of *C. rosea* (“golden ring”) compared with those of its original cultivar (“pumpkin pie”) and the commercially available coreopsis tea (“snow chrysanthemum”) as control groups (Figure 1), via gas chromatography-mass spectrometry (GC-MS). Their antioxidant activities were also evaluated using 1,1-diphenyl-2-picrylhydraxyl (DPPH) and 2,2-azino-bis-3-ethylbenzothiazoline-6-sulfonic acid (ABTS) radical scavenging assays.

## 2. Results and Discussion

### 2.1. Chemical Composition of Dichloromethane Extracts of Three Different Coreopsis Cultivars

The flowers of “pumpkin pie”, “golden ring” and “snow chrysanthemum” were extracted with dichloromethane. The volatile components of these flowers were identified based on their molecular formula, retention index (RI) and National Institute of Standards and Technology (NIST) library similarity index. The GC-MS analysis revealed a total of 89 components in these flowering materials, which led to the identification of 50 components in “snow chrysanthemum”, 58 in “pumpkin pie” and 59 in “golden ring” (Table 1).

**Figure 2:**
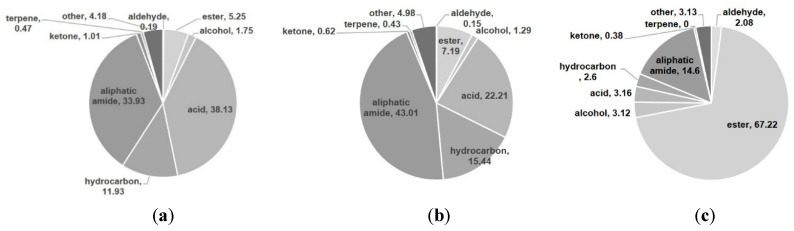
Relative content (%) of volatile compounds in (**a**) “golden ring”; (**b**) “pumpkin pie”; (**c**) “snow chrysanthemum”.

**Figure 3:**
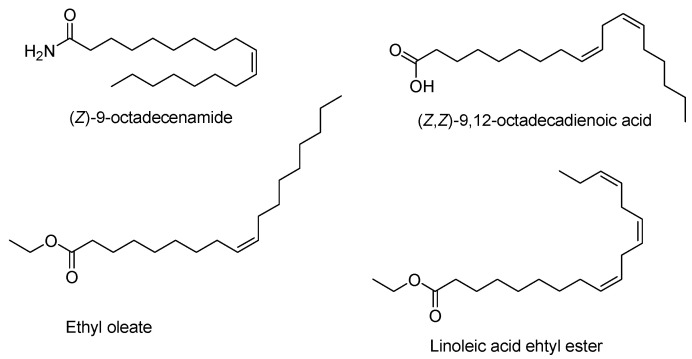
The chemical structures of the most intense components in “golden ring”, “pumpkin pie” and “snow chrysanthemum”.

The volatile compounds were classified into seven classes: hydrocarbons, fatty acids, alcohols, ketones, esters, terpenoids and aliphatic amides (Figure 2). Table 2 summarizes the ten most abundant constituents among the volatile compounds detected via GC-MS. The volatile oils of “golden ring” and “pumpkin pie” comprised a large proportion of aliphatic amides, fatty acids and hydrocarbons. “Golden ring” contained total volatile oils constituting 96.84% of its total volatile compounds, including 38.13% fatty acids, 33.93% aliphatic amides and 11.263% hydrocarbons, while “pumpkin pie” contained 43.01% aliphatic amides, 22.21% fatty acids and 15.44% hydrocarbons (Figure 2). The extract of “snow chrysanthemum” comprised total volatile compounds up to 96.29%, including esters (67.22%), aliphatic amides (14.6%) and acids (3.16%) (Figure 2). Table 3 summarizes the ten most abundant compounds among the volatile components of these cultivars detected via GC-MS. The major compound in the “golden ring” extract was (*Z*)-9-octadecenamide constituting 28.38% of the total composition (Figure 3), followed by (*Z,Z*)-9,12-octadecadienoic acid (17.36%), *n*-hexadecanoic acid (7.83%), (*Z,Z,Z*)-9,12,15-octadecatrienoic acid (6.86%) and nonacosane (3.88%) (Table 2). The major compound in the “pumpkin pie” extract was also (*Z*)-9-octadecenamide (37.48%), followed by (*Z,Z*)-9,12-octadecadienoic acid (8.69%), heptacosane (5.06%), nonacosane (4.67%) and (*Z,Z,Z*)-9,12,15-octadecatrienoic acid (4.65%). In contrast to the volatile oils of “golden ring” and “pumpkin pie”, the main constituent of “snow chrysanthemum” was ethyl oleate (28.53%) (Figure 3), followed by linoleic acid ethyl ester (16.89%), hexadecenoic acid ethyl ester (14.0%), (*Z*)-9-octadecenamide (12.57%) and (*E*)-9-octadecenoic acid ethyl ester (2.07%) (Table 2).

Reports have suggested that (*Z*)-9-octadecenamide is predominant in the natural volatile oil derived from “golden ring” and “pumpkin pie”, with strong antioxidant and antimicrobial properties [16]. In addition, extracts containing (*Z,Z*)-9,12-octadecadienoic acid (linoleic acid) and (*Z,Z,Z*)-9,12,15-ocatadecadienoic acid (linolenic acid), which are abundant in “golden ring” and “pumpkin pie” but less abundant in “snow chrysanthemum”, have also been reported to exhibit antioxidant activities [17,18]. It has been reported that *n*-hexadecanoic acid (palmitic acid), which is relatively high in “golden ring”, has anti-inflammatory [19,20] and anti-cancer effects [21]. The high content of volatile esters in “snow chrysanthemum” may contribute to the strong flavor of the tea.

### 2.2. Antioxidant Activities of Dichloromethane Extracts Obtained from Three Different Coreopsis Cultivars

The antioxidant activity of dichloromethane extracts of the three different *Coreopsis* cultivars was evaluated via ABTS and DPPH radical scavenging assays. These are rapid and sensitive methods used to evaluate antioxidant effects due to the hydrogen donor properties of the substance. The “golden ring” extract showed higher DPPH radical scavenging activity—with a 50% inhibitory concentration (IC_50_) value of 602.1 μg/mL—than did “pumpkin pie” (IC_50_ 2291.3 μg/mL) or “snow chrysanthemum” (IC_50_ 3166.2 μg/mL). As in the DPPH free radical scavenging activities, the “golden ring” extract showed optimal antioxidant activity with an IC_50_ of 137.0 μg/mL in the ATBS radical scavenging assay (Table 3). Previous studies have reported the biological activities of the fatty acids in “golden ring”, such as anti-oxidant activities [22,23,24], cellular reactive oxygen species generation [25] and anti-cancer activity [26]. Thus, the potent antioxidant activity of the volatile extract of “golden ring” is attributed to the twofold higher fatty acid content compared with that of “pumpkin pie”.

## 3. Materials and Methods

### 3.1. Plant Material

Radiation mutants of *C. resae*, “golden ring”, were generated via the treatment of stem cuttings of the original cultivars, “pumpkin pie”, with gamma (^60^Co) irradiation (150 TBq capacity; AECL, Ottawa, ON, Canada). These mutants were selected from variants in floral size and showed stable inheritance of the phenotype for four years. The radiation mutant cultivars were grown by Uriseed Group Corporation (Icheon-si, Gyeonggi-do, Korea) and registered as new plant varieties in the Korea Seed and Variety Service. The flowers were handpicked and randomly collected at the flowering stage in the same plantation. They were freeze-dried and stored at −20 °C in polyethylene plastic bags until further analysis. Voucher specimens were deposited at the Uriseed Group Corporation. “Snow chrysanthemum” was purchased in China as a commercially available tea material.

### 3.2. Sample Preparation

Fresh flowers of “golden ring” and “pumpkin pie” were freeze-dried and ground into powder. The dried tea material from “snow chrysanthemum” was ground into powder. Samples (200 mg each) were directly extracted with 20 mL of dichloromethane in an ultrasonic bath for 60 min. Subsequently, the extracts were dehydrated over 0.5 g of anhydrous sodium sulfate and filtered through a polyvinylidene fluoride syringe filter (0.45 µm) for GC-MS analysis.

### 3.3. GC-MS Analysis

Dichloromethane extracts were analyzed using a Shimadzu QP-2010 Ultra (Shimadzu, Kyoto, Japan). The compounds were separated on the DB-5MS capillary column (30 m × 0.25 mm × 0.25 µm; Agilent Technologies Co., Santa Clara, CA, USA). The carrier gas was 99.99% high-purity helium with a column flow rate of 1.53 mL/min and the injection port in splitless mode. The oven temperature was 50 °C, which was gradually increased to 100 °C at a rate of 5 °C/min and held steady for 5 min, then increased to 230 °C at a rate of 5 °C/min. After holding steady for 20 min again, it was finally increased to 250 °C at a rate of 10 °C/min, and held steady for 5 min. The MS parameters were electron ionization (EI) mode with ionization voltage 70 eV, ion source temperature 230 °C and scan range 45–450 *m/z*.

The retention indices of all GC peaks were calculated using the retention times of C7–C30 n-alkane standards under the same chromatographic conditions (Figures S1 and S2). Each peak was identified by comparing with the mass spectra database (National Institute of Standards and Technology, Mass Spectra Libraries, Gaithersburg, MD, USA) [27,28].

(1) $$I=100\times \left(\frac{T_{i}-T_{n}}{T_{n+1}-T_{n}}+n\right)$$

In Equation (1) above, *I* denotes the retention index of the components to be tested and i is the adjusted retention time (min) of the components to be tested; n and n + 1 represent the carbon amounts of n-alkanes before and after the diffusion of unknown substances, respectively. The values of T_n_ and T_n+1_ denote the carbon retention times of n-alkanes.

### 3.4. Measurement of DPPH Free Radical and ABTS Radical Cation Scavenging Activities

The DPPH of each sample was determined using Brand-Williams’ method [29]. Briefly, the 2 mM DPPH solution was diluted with ethanol to an absorbance of less than 1.0 at 517 nm before the analysis. Each dichloromethane extract was evaporated in vacuo and initially dissolved in dimethyl sulfoxide (DMSO) to produce 5 mg/mL stock solution. Subsequently, the final concentrations of the samples were diluted to 125, 250, 1000 and 5000 μg/mL using DMSO. Next, each sample was suspended in DMSO and 40 μL of the sample was made to react with 160 μL of 0.2 mM DPPH solution. After 6 min, absorbance was measured at 517 nm using an ELISA reader (Benchmark Plus, Bio-Rad, Hercules, CA, USA).

The ABTS of each sample was determined using the method published by Re et al. [30]. In brief, the ABTS was measured with a pre-formed radical monocation. The mixtures, along with 7.4 mM ABTS solution and 2.6 mM potassium persulfate, were incubated at room temperature in the dark for 24 h. The ABTS solution was diluted with phosphate-buffered saline (pH 7.4) to achieve an absorbance of 0.7 ± 0.02 at 734 nm. The final concentrations of the extracts were diluted to 25, 125, 250 and 500 μg/mL using DMSO and 40 μL of the sample was made to react with 160 μL of the ABTS solution. Absorbance was measured 6 min after the reaction at 734 nm using an ELISA reader (Benchmark Plus, Bio-Rad, Hercules, CA, USA). The DPPH free radical and ABTS radical scavenging activities (%) were calculated as follows:

(2) $$\mathrm{Scavenging}\;\mathrm{effect}\left(\%\right)=1-\left(\frac{\mathrm{Asample}}{\mathrm{Acontrol}}\times 100\right)$$

where Asample and Acontrol represent the absorbance of the sample and control, respectively.

The 50% inhibitory concentration (IC_50_) of the extract was calculated using a linear standard curve, and the IC_50_ of ascorbic acid, which was used as a positive control, was determined on the basis of its molecular mass.

## 4. Conclusions

The volatile compounds obtained from the dichloromethane extract of the gamma-irradiated mutant cultivar of *C. rosea* (“golden ring”), its original cultivar (“pumpkin pie”) and the tea commercially available in China (*C. tinctoria*; “snow chrysanthemum”) were successfully identified by GC-MS. The major components of “golden ring” and “pumpkin pie” varied slightly, although along with “snow chrysanthemum” they showed significant differences in chemical composition. Meanwhile, the antioxidant effect of “golden ring” extract was higher compared with that of “pumpkin pie” and “snow chrysanthemum”. Despite phytochemical and pharmacological studies of *C. tinctoria*, the chemical composition and antioxidant activity of *C. rosea* was reported for the first time in this study. The chemical composition and strong antioxidant efficacy of “golden ring” favor the development of tea materials such as “snow chrysanthemum”.

## Figures and Tables

**Table 1:** Composition of volatile compounds of the dichloromethane extracts obtained from “golden ring”, “pumpkin pie” and “snow chrysanthemum”.

No.	Compound	Formula	RI ^1^	%Relative		
				Golden Ring	Pumpkin Pie	Snow Chrysanthemum
1	Nonanal	C_9_H_18_O	1104	-	0.02	-
2	Octanoic acid ethyl ester	C_10_H_20_O_2_	1195	-	-	0.38
3	(*E*)-2-Decenal	C_10_H_18_O	1263	0.03	0.03	0.51
4	2,4-Decadienal	C_10_H_16_O	1296	-	-	0.17
5	(*E*,*E*)-2,4-Decadienal	C_10_H_16_O	1319	0.04	0.03	0.21
6	1-Methyl-4-(1-methylethenyl)-1,2-cyclohexanediol	C_10_H_18_O_2_	1347	-	-	0.46
7	n-Decanoic acid	C_12_H_24_O_2_	1356	0.28	0.05	-
8	2,4,7,9-Tetramethyl-5-decyn-4,7-diol	C_14_H_26_O_2_	1405	0.1	0.07	-
9	2,4-Bis(1,1-dimethylethyl)phenol	C_14_H_24_O_2_	1504	0.03	-	-
10	9-Oxo-nonanoic acid ethyl ester	C_11_H_20_O_3_	1500	-	-	0.37
11	5,6,7,7a-Tetrahydro-4,4,7a-trimethyl-2(4*H*)-benzofuranone	C_11_H_16_O_2_	1535	0.05	-	-
12	Dodecanoic acid	C_12_H_24_O_2_	1556	0.67	0.13	0.13
13	Fumaric acid ethyl 2-methylallyl ester	C_10_H_14_O_4_	1562	0.05	-	-
14	Hexadecane	C_16_H_34_	1599	-	-	-
15	Farnesene epxide	C_15_H_24_O	1610	-	0.03	-
16	Fluorene	C_13_H_10_	1644	0.06	0.02	-
17	(*Z*)-9-Tetradecenal	C_14_H_26_O	1659	-	-	0.89
18	Aromadendren oxide	C_15_H_24_O	1689	0.06	0.06	-
19	(*Z*)-*trans*-*α*-Bergamotol	C_15_H_24_O	1696	0.12	0.18	-
20	Heptadecane	C_17_H_36_	1699	0.03	-	-
21	Fluorenone	C_13_H_8_O	1709	0.09	0.03	-
22	Fluorene-4-carboxylic acid	C_14_H_12_O_2_	1728	-	0.09	-
23	Methyl trans-*p*-coumarate	C_10_H_10_O_3_	1742	-	0.05	-
24	2-Methyl-5-(1,2,2-trimethylcyclopentyl)phenol	C_15_H_22_O	1745	0.15	0.05	-
25	Tetradecanoic acid	C_14_H_28_O_2_	1754	0.46	0.43	0.2
26	(*Z*)-7-Hexadecenal	C_16_H_30_O	1761	-	-	0.07
27	Tetradecanoic acid ethyl ester	C_16_H_32_O_2_	1790	-	-	0.18
28	Octadecane	C_18_H_38_	1799	-	0.04	-
29	Cyclopentadecanon	C_15_H_28_O	1830	-	-	0.12
30	6,10,14-Trimethyl-2-pentadecanone	C_18_H_36_O	1838	0.16	0.06	0.08
31	Pentadecanoic acid	C_15_H_30_O_2_	1853	0.12	0.06	-
32	(*Z*)-9-Hexadecen-1-ol	C_16_H_32_O	1878	0.04	0.03	-
33	7,9-Di-tert-butyl-1-oxaspiro(4,5)deca-6,9-diene-2,8-dione	C_17_H_34_O_3_	1901	0.08	0.08	-
34	1,2-Dihydro-5-acenaphthylenecarboxaldehyde	C_12_H_9_NO_2_	1947	1.3	0.78	-
35	*n*-Hexadecanoic acid	C_16_H_32_O_2_	1961	7.83	3.87	1.42
36	Tetradecanamide	C_14_H_29_NO	1967	0.63	0.5	0.33
37	Ethyl 9-hexadecenoate	C_18_H_34_O_2_	1967	-	-	0.11
38	Hexadecanoic acid ethyl ester	C_18_H_36_O_2_	1991	-	-	14
39	11-Hexadecyn-1-ol	C_18_H_34_O_2_	1995	-	-	0.14
40	(*Z*)-9,17-Octadecadienal	C_18_H_32_O	2028	0.12	0.07	0.23
41	(*Z,Z*)-9,12-Octadecadien-1-ol	C_18_H_34_O	2048	0.07	0.03	-
42	9,12-Octadecadienoic acid methyl ester	C_19_H_34_O_2_	2087	0.05	-	-
43	Phytol	C_20_H_40_O	2105	0.29	0.16	-
44	(*Z,Z*)-9,12-Octadecadienoic acid	C_18_H_32_O_2_	2135	17.36	8.69	0.86
45	(*Z,Z,Z*)-9,12,15-Octadecatrienoic acid	C_18_H_30_O_2_	2139	6.86	4.65	0.35
46	Linoleic acid ethyl ester	C_20_H_36_O_2_	2155	-	-	16.89
47	17-Octadecynoic acid	C_18_H_30_O_2_	2158	2.59	2.93	-
48	Octadecanoic acid	C_18_H_36_O_2_	2160	1.96	1.4	-
49	Ethyl oleate	C_18_H_38_O_2_	2165	-	-	28.53
50	(*E*)-9-Octadecenoic acid ethyl ester	C_18_H_38_O_2_	2170	-	-	2.07
51	Hexadecanamide	C_16_H_33_NO	2175	2.11	2.51	0.1
52	Octadecanoic acid ethyl ester	C_20_H_40_O_2_	2190	-	-	1.67
53	Tricosane	C_23_H_48_	2299	0.33	0.31	0.35
54	(*Z*,*Z*)-11,14-Eicosadienoic acid methyl ester	C_21_H_38_O_2_	2334	0.26	-	-
55	Isopropyl linoleate	C_21_H_38_O_2_	2344	2.63	2.69	1.3
56	(*Z*)-9-Octadecenamide	C_18_H_35_NO	2363	28.38	37.48	12.57
57	Octadecanamide	C_18_H_36_NO	2381	0.54	0.56	1.5
58	PGH1 methyl ester	C_22_H_38_O_2_	2384	-	-	0.23
59	Eicosanoic acid ethyl ester	C_22_H_44_O_2_	2390	-	-	0.72
60	Tetracosane	C_24_H_50_	2399	0.2	0.28	0.18
61	13-Docosenoic acid	C_22_H_44_O_2_	2401	-	-	0.2
62	1,22-Docosanediol	C_22_H_46_O_2_	2413	-	-	1.55
63	1-Heptadec-1-ynyl-cyclohexanol	C_23_H_42_O	2425	-	-	0.12
64	Behenic alcohol	C_22_H_46_O	2488	0.39	0.26	0.4
65	Pentacosane	C_25_H_52_	2499	1.96	2.64	0.76
66	2-Hydroxy-1-(hydroxymethyl)ethyl palmitate	C_19_H_38_O_4_	2503	1.47	2.24	-
67	Bis(2-ethylhexyl) phthalate	C_24_H_38_O_4_	2523	0.53	1.08	0.23
68	(*Z*)-11-Eicosenamide	C_20_H_39_NO	2559	0.19	0.66	0.1
69	3,6-Nonadecadione	C_19_H_36_O_2_	2583	0.85	0.56	0.3
70	Ethyl docosanoate	C_24_H_48_O_2_	2591	-	-	0.23
71	Hexacosane	C_26_H_54_	2599	0.34	0.48	-
72	Tricosyl acetate	C_25_H_50_O_2_	2681	-	0.56	-
73	Pentacosanol	C_25_H_52_O	2693	0.89	-	0.45
74	Heptacosane	C_27_H_56_	2701	3.8	5.06	1.36
75	Ethyl tetracosanoate	C_26_H_52_O_2_	2705	-	-	0.31
76	2,3-Dihydroxypropyl stearate	C_21_H_42_O_4_	2711	0.23	0.94	-
77	1-Pentacosanol	C_25_H_52_O	2722	0.26	0.9	-
78	Di-n-octylphthalate	C_24_H_38_O_4_	2732	-	0.2	-
79	13-Docosenamide	C_22_H_43_NO	2767	0.78	0.52	-
80	Octacosane	C_28_H_58_	2798	0.48	0.72	-
81	2-Methyloctacosane	C_29_H_60_	2898	0.19	-	1.72
82	Nonacosane	C_29_H_60_	2901	3.88	4.67	-
83	Hexacosanoic acid methyl ester	C_27_H_54_O_2_	2928	0.08	0.19	-
84	Hentriacontane	C_31_H_64_	-	1.24	1.55	0.3
85	Vitamin E	C_29_H_50_O_2_	-	0.25	0.25	-
86	Campesterol	C_28_H_48_O	-	0.18	0.2	-
57	Stigmasterol	C_29_H_48_O	-	1.04	1.29	0.26
88	*γ*-Sitosterol	C_29_H_50_O	-	0.68	0.99	0.33
89	*α*-Amyrin	C_30_H_50_O	-	1	0.91	0.35
				96.84	95.32	96.29

^1^ RI: retention index.

**Table 2:** Top ten volatile compounds in “golden ring”, “pumpkin pie” and “snow chrysanthemum”.

No.	Golden Ring	Pumpkin Pie	Snow Chrysanthemum
1	(*Z*)-9-Octadecenamide (28.38%)	(*Z*)-9-Octadecenamide (37.48%)	Ethyl oleate (28.53%)
2	(*Z,Z*)-9,12-Octadecadienoic acid (17.36%)	(*Z,Z*)-9,12-Octadecadienoic acid (8.69%)	Linoleic acid ethyl ester (16.89%)
3	*n*-Hexadecanoic acid (7.83%)	Heptacosane (5.06%)	Hexadecanoic acid ethyl ester (14.0%)
4	(*Z,Z,Z*)-9,12,15-Octadecadienoic acid (6.86%)	Nonacosane (4.67%)	(*Z*)-9-Octadecenamide (12.57%)
5	Nonacosane (3.88%)	(*Z,Z,Z*)-9,12,15-Octadecadienoic acid (4.65%)	(*E*)-9-Octadecenoic acid ethyl ester (2.07%)
6	Heptacosane (3.80%)	*n*-Hexadecanoic acid (3.87%)	2-Methyloctacosane (1.72%)
7	Isopropyl linoleate (2.63%)	17-Octadecynoic acid (2.93%)	Ethyl octadecanoate (1.67%)
8	17-Octadecynoic acid (2.59%)	Isopropyl linoleate (2.69%)	1,22-Docosanediol (1.55%)
9	Hexadecamide (2.11%)	Pentacosane (2.64%)	Octadecamamide (1.50%)
10	Octadecanoic acid (1.96%) Pentacosane (1.96%)	Hexadecamide (2.51%)	*n*-Hexadecanoic acid (1.42%)

**Table 3:** Antioxidant activities of the dichloromethane extract of three different *Coreopsis* cultivars.

Sample	ABTS ^1^ (IC_50_, μg/mL)^2^	DPPH ^1^ (IC_50_, μg/mL)
Golden ring (mutant cultivar)	137.0	602.1
Pumpkin pie (original cultivar)	262.8	2291.3
Snow chrysanthemum (tea material)	278.4	3166.2
Ascorbic acid (positive control)	29.3 μM	100.9 μM

^1^ ABTS: 2,2-azino-bis-3-ethylbenzothiazoline-6-sulfonic acid; DPPH: 1,1-diphenyl-2-picrylhydraxyl. ^2^ IC50: 50% inhibition of concentration.

## Supplementary Materials

The following are available online at https://www.mdpi.com/2223-7747/9/6/717/s1: Figure S1: GC-MS chromatograms of the dichloromethane extract of (a) “golden ring”, (b) “pumpkin pie” and (c) “snow chrysanthemum”; Figure S2: GC-MS chromatograms of n-alkane standard.
