# Supplementary material for: Composition and Antioxidant Activities of Volatile Organic Compounds in Radiation-Bred Coreopsis Cultivars
Source: Plants (Basel). 2020 Jun 4;9(6):717. doi: 10.3390/plants9060717 (PMC7356690; doi:10.3390/plants9060717)
Supplement: Supplementary file 1 [file plants-09-00717-s001.pdf]

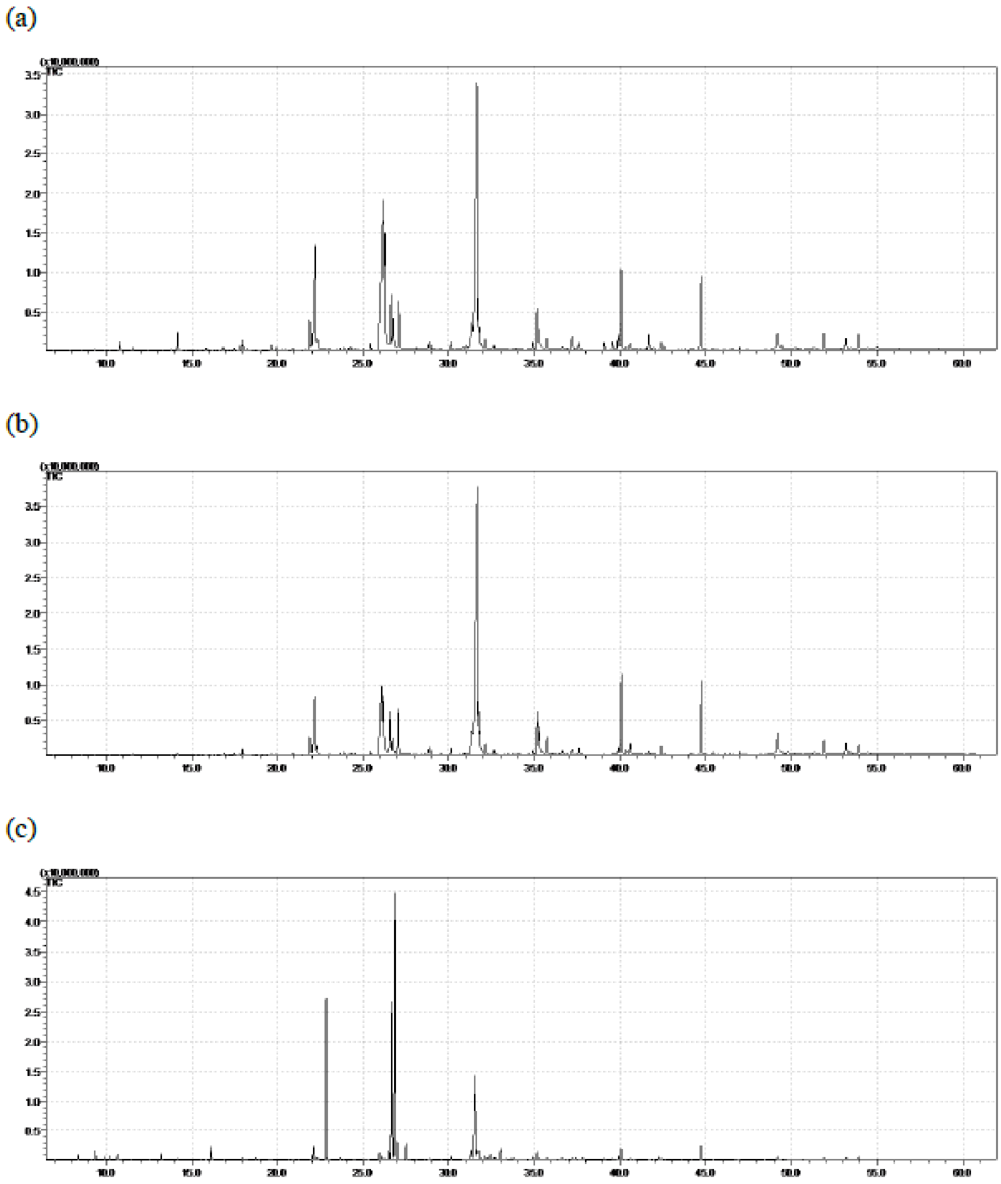

Figure S1. GC-MSchromatograms of the dichloromethane extractof (**a**) 'golden ring',(**b**)'pumpkin pie' and (**c**) 'snow chrysanthemum'.

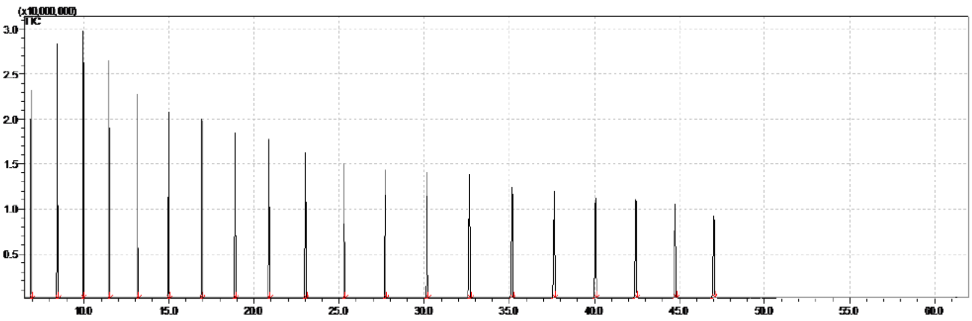

Figure S2. GC-MSchromatograms of n-alkane standard.
